# Plasma neurofilament light chain and amyloid-β are associated with the kynurenine pathway metabolites in preclinical Alzheimer’s disease

**DOI:** 10.1186/s12974-019-1567-4

**Published:** 2019-10-10

**Authors:** Pratishtha Chatterjee, Henrik Zetterberg, Kathryn Goozee, Chai K. Lim, Kelly R. Jacobs, Nicholas J. Ashton, Abdul Hye, Steve Pedrini, Hamid R. Sohrabi, Tejal Shah, Prita R. Asih, Preeti Dave, Kaikai Shen, Kevin Taddei, David B. Lovejoy, Gilles J. Guillemin, Kaj Blennow, Ralph N. Martins

**Affiliations:** 1 Department of Biomedical Sciences, Macquarie University, North Ryde, NSW Australia; 2 School of Medical Health and Sciences, Edith Cowan University, Joondalup, WA Australia; 3 Department of Psychiatry and Neurochemistry, Institute of Neuroscience and Physiology, University of Gothenburg, Mölndal, Sweden; 4 Clinical Neurochemistry Laboratory, Sahlgrenska University Hospital, Mölndal, Sweden; 5 Department of Neurodegenerative Disease, UCL Institute of Neurology, Queen Square, London, UK; 6 UK Dementia Research Institute at UCL, London, UK; 7 KaRa Institute of Neurological Disease, Sydney, Macquarie Park, NSW Australia; 8 Clinical Research Department, Anglicare, Sydney, Castle Hill, NSW Australia; 9 School of Psychiatry and Clinical Neurosciences, University of Western Australia, Crawley, WA Australia; 10 Institute of Psychiatry, Psychology and Neuroscience, Maurice Wohl Institute Clinical Neuroscience Institute, King’s College London, London, UK; 11 NIHR Biomedical Research Centre for Mental Health and Biomedical Research Unit for Dementia, South London and Maudsley NHS Foundation, London, UK; 12 Wallenberg Centre for Molecular and Translational Medicine, University of Gothenburg, Gothenburg, Sweden; 13 Australian Alzheimer’s Research Foundation, Nedlands, WA Australia; 14 Australian eHealth Research Centre, CSIRO, Floreat, WA Australia; 15 The Cooperative Research Centre for Mental Health, Carlton South, VIC Australia; 16 School of Medical and Health Sciences, Edith Cowan University, Ralph & Patricia Sarich Neuroscience Research Institute, 8 Verdun Street, Nedlands, WA 6009 Australia

**Keywords:** Blood markers, Neuroinflammation, Neurodegeneration, Alzheimer’s disease, Neurofilament light chain, Amyloid-beta, Kynurenine pathway, Brain amyloid-beta, Blood amyloid-beta

## Abstract

**Background:**

Blood markers indicative of neurodegeneration (neurofilament light chain; NFL), Alzheimer’s disease amyloid pathology (amyloid-β; Aβ), and neuroinflammation (kynurenine pathway; KP metabolites) have been investigated independently in neurodegenerative diseases. However, the association of these markers of neurodegeneration and AD pathology with neuroinflammation has not been investigated previously. Therefore, the current study examined whether NFL and Aβ correlate with KP metabolites in elderly individuals to provide insight on the association between blood indicators of neurodegeneration and neuroinflammation.

**Methods:**

Correlations between KP metabolites, measured using liquid chromatography and gas chromatography coupled with mass spectrometry, and plasma NFL and Aβ concentrations, measured using single molecule array (Simoa) assays, were investigated in elderly individuals aged 65–90 years, with normal global cognition (Mini-Mental State Examination Score ≥ 26) from the Kerr Anglican Retirement Village Initiative in Ageing Health cohort.

**Results:**

A positive correlation between NFL and the kynurenine to tryptophan ratio (K/T) reflecting indoleamine 2,3-dioxygenase activity was observed (*r* = .451, *p* < .0001). Positive correlations were also observed between NFL and kynurenine (*r* = .364, *p* < .0005), kynurenic acid (*r* = .384, *p* < .0001), 3-hydroxykynurenine (*r* = .246, *p* = .014), anthranilic acid (*r* = .311, *p* = .002), and quinolinic acid (*r* = .296, *p* = .003). Further, significant associations were observed between plasma Aβ40 and the K/T (*r* = .375, *p* < .0005), kynurenine (*r* = .374, *p* < .0005), kynurenic acid (*r* = .352, *p* < .0005), anthranilic acid (*r* = .381, *p* < .0005), and quinolinic acid (*r* = .352, *p* < .0005). Significant associations were also observed between plasma Aβ42 and the K/T ratio (*r* = .215, *p* = .034), kynurenic acid (*r* = .214, *p* = .035), anthranilic acid (*r* = .278, *p* = .006), and quinolinic acid (*r* = .224, *p* = .027) in the cohort. On stratifying participants based on their neocortical Aβ load (NAL) status, NFL correlated with KP metabolites irrespective of NAL status; however, associations between plasma Aβ and KP metabolites were only pronounced in individuals with high NAL while associations in individuals with low NAL were nearly absent.

**Conclusions:**

The current study shows that KP metabolite changes are associated with biomarker evidence of neurodegeneration. Additionally, the association between KP metabolites and plasma Aβ seems to be NAL status dependent. Finally, the current study suggests that an association between neurodegeneration and neuroinflammation manifests in the periphery, suggesting that preventing cytoskeleton cytotoxicity by KP metabolites may have therapeutic potential.

**Electronic supplementary material:**

The online version of this article (10.1186/s12974-019-1567-4) contains supplementary material, which is available to authorized users.

## Introduction

Neurofilament light chain (NFL), an axonal cytoskeletal protein, is an emerging blood marker for neurodegenerative diseases, for review see [1]. Since neurodegenerative processes involve axonal damage resulting in the release of neuronal NFL into extracellular spaces with subsequent diffusion into the cerebrospinal fluid (CSF) and the blood, elevated CSF and blood NFL concentrations are now recognised as features of several neurodegenerative diseases [2–7], including Alzheimer’s disease (AD) [8].

**Fig. 1:**
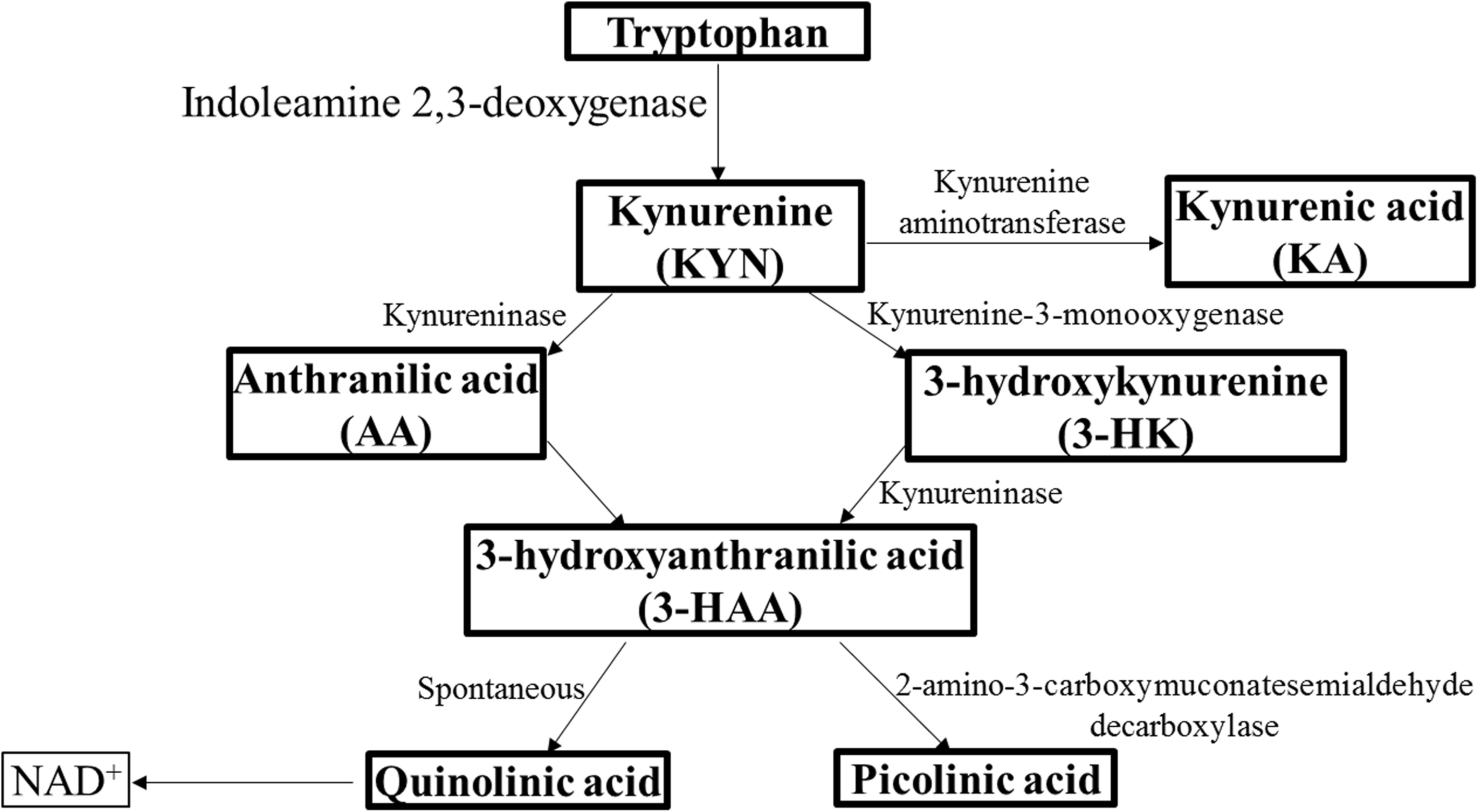
The kynurenine pathway schematic. Within the KP, tryptophan is metabolised to kynurenine (KYN) via the enzymes indoleamine 2,3-deoxygenase or tryptophan deoxygenase and formamidase. KYN gets converted to kynurenic acid (KA) via the enzyme kynurenine aminotransferase. KYN is also metabolised to anthranilic acid (AA) by the enzyme kynureninase and to 3-hydroxykynurenine (3-HK) by the enzyme kynurenine-3-monooxygenase. AA and 3-HK are metabolised to 3-hydroxyanthranilic acid (3-HAA). 3-HAA further converts to aminocarboxymuconic semialdehyde that spontaneously either converts to the neurotoxin, quinolinic acid, a substrate for the redox agent, NAD+ or is assisted by enzyme 2-amino-3-carboxymuconatesemialdehyde decarboxylase to generate picolinic acid

Neurodegeneration is known to be associated with neuroinflammation [9], and the kynurenine pathway (KP) is strongly upregulated in neuroinflammatory conditions [10–13]. The KP is induced by the activation of the enzyme, indoleamine 2,3-dioxygenase (IDO1), resulting in the metabolism of tryptophan (TRP) to kynurenine (KYN). Following the formation of KYN, the enzyme kynurenine aminotransferase converts KYN to kynurenic acid (KA). Alternatively, the enzymes kynureninase and kynurenine 3-monooxygenase metabolise KYN to anthranilic acid (AA) and 3-hydroxykynurenine (3-HK), respectively. Further, AA and 3-HK get metabolised to 3-hydroxyanthranilic acid (3-HAA), which further converts to aminocarboxymuconic semialdehyde (ACMA). ACMA either generates picolinic acid (PA) via the enzyme 2-amino-3-carboxymuconatesemialdehyde decarboxylase or spontaneously gets converted to quinolinic acid (QA), a neurotoxin and precursor for the redox agent, nicotinamide adenine dinucleotide (Fig. 1) [14]. Several inflammatory molecules such as interferon-γ, tumour necrosis factor-α, toll-like receptor 4, and lipopolysaccharide activate IDO1 triggering the production of several neuroactive KP metabolites by most of the brain cells, i.e. microglia, astrocytes, neurons, and infiltrating macrophages [15].

Increasing evidence shows that the KP is triggered in several neurodegenerative diseases [10, 16–19]. Further, several studies including those from our laboratory also report associations between the KP metabolites and AD pathogenesis, from the preclinical stage prior to cognitive decline to the clinical stage [14, 20–23]. For instance, higher plasma KYN to TRP (K/T) ratios, reflecting higher IDO activity, have been observed within the different stages of AD pathogenesis [14, 24, 25]. Two relatively recent studies observed elevated plasma AA concentrations in preclinical AD and in individuals at risk to dementia [14, 26]. Additionally, a decline in serum KA [27] and increase in plasma QA [28] have been observed in individuals with mild cognitive impairment (MCI) and AD.

While NFL and the KP have both independently been reported to be associated with neurodegenerative diseases as described above, the association between the neurodegenerative marker, NFL, and markers indicative of neuroinflammation, such as KP metabolites, has not been investigated previously. The current study therefore investigated whether an association between NFL and KP metabolites was present in cognitively normal elderly individuals to provide insight on the association between blood indicators of neurodegeneration and neuroinflammation. Further, the increasing emphasis of plasma amyloid-β (Aβ) as a potential marker for AD [29, 30] led us to also investigate the associations between KP metabolites and plasma Aβ 40 and 42 species within the current study. Additionally, participants within the current study were also categorised as those within the preclinical phase of AD, characterised by high neocortical Aβ load (NAL) assessed via positron emission tomography (PET), and those with no apparent risk to AD or low NAL, to separately evaluate the aforementioned associations in each group (high/low NAL) in order to assess whether these associations were specific to AD-related neuropathology.

## Methods

### Participants

Participants from the Kerr Anglican Retirement Village Initiative in Ageing Health (KARVIAH) cohort, at baseline, described previously [31], were utilised in the current study. Briefly, 134 volunteers met the set screening eligibility inclusion and exclusion criteria for the KARVIAH cohort. The inclusion criteria comprised an age range of 65–90 years, good general health, no known significant cerebral vascular disease, fluent in English, adequate/corrected vision and hearing to enable testing, and no objective cognitive impairment as screened by a Montreal Cognitive Assessment (MoCA; a 0–30 point test assessing cognitive domains including short-term recall, learning trials, delayed recall, visuospatial abilities, three-dimensional cube, trail making, fluency, abstraction, and attention) score ≥ 26. MoCA scores lying between 18 and 25 were assessed on a case by case basis by the study neuropsychologist following stratification of scores according to age and education [32]. The exclusion criteria comprised the diagnosis of dementia based on the revised criteria from the National Institute on Aging-Alzheimer’s Association [33], presence of acute functional psychiatric disorder (including lifetime history of schizophrenia or bipolar disorder), history of stroke, severe or extremely severe depression (based on the Depression Anxiety Stress Scales), and uncontrolled hypertension (systolic BP > 170 mmHg or diastolic BP > 100 mmHg).

The 105 participants who met the eligibility criteria underwent neuroimaging, neuropsychometric evaluation, and blood collection, while the remaining either withdrew or declined neuroimaging. Within these 105 participants, 100 participants were considered to have normal global cognition based on their Mini-Mental State Examination (a 0–30 point test used to screen cognitive impairment) score (MMSE ≥ 26) [34] and were included in the current study. Plasma NFL and KP concentrations were measured in all 100 participants with MMSE ≥ 26, while plasma Aβ 40 and 42 species were measured in 98 and 97 participants (95 common), respectively, of the 100 participants with MMSE ≥ 26. All volunteers provided written informed consent prior to participation, and the Bellberry Human Research Ethics Committee, Australia (reference no. 2012-09-1086), and the Macquarie University Human Research Ethics Committee, Australia, provided approval for the study (reference no. 5201701078).

### Blood collection and APOE genotyping

All participants fasted for a minimum of 10 h overnight prior to blood withdrawal employing standard serological methods and processing of serum and plasma [35]. Apolipoprotein E (*APOE*) genotype was determined from purified genomic DNA extracted from 0.5 mL whole blood as previously described [35].

### Measurement of plasma neurofilament light chain and amyloid-β concentrations

The ultra-sensitive single molecule array (Simoa, Quanterix) platform was employed to measure plasma NFL and Aβ concentrations [8, 36, 37]. Calibrators were run in duplicates while samples were diluted fourfold for NFL and Aβ42, and eightfold for Aβ40 and run in singlicates. Two quality control (QC) samples were run in duplicates at the beginning and end of each plate for NFL, wherein a QC sample with a concentration of 12.1 pg/mL had repeatability and intermediate precision of 20.2%, while for a QC sample with a concentration of 155.8 pg/mL, repeatability was 14.6% and intermediate precision was 14.9%. For Aβ40, a QC sample with a concentration of 219 pg/mL, repeatability was 6.9% and intermediate precision was 7.9%. For Aβ42, a QC sample with a concentration of 12.9 pg/mL, repeatability was 2.4% and intermediate precision was 5.6%.

### Measurement of serum kynurenine pathway metabolites

Serum samples were collected in 2015 and stored at − 80 °C until thawed for measurement of KP metabolites in 2017. Serum kynurenine metabolites were measured as described previously [14]. Briefly, analyses of tryptophan, KYN, 3-HK, 3-HAA, and AA were performed simultaneously with ultra-high-performance liquid chromatography (UHPLC) [11]. KA separation was carried out following injection (10 μL) onto a Poroshell RRHT C-18, 1.8 μm 2.1 × 100 mm column (Agilent Technologies, Inc., Santa Clara, CA), and quantified via fluorescence detection (excitation and emission wavelengths of 344 nm and 388 nm, respectively; retention time 1.5 min). A gradient method with 50 mM sodium acetate, 25 mM zinc acetate and 2.25% acetonitrile (eluent A), and 10% acetonitrile (eluent B) was employed, and separation was performed at 38 °C with a flow rate of 0.75 mL/min. Separation achieved over 10 min comprised 100% eluent A for 3 min, 50% eluent A for 2 min, 0% eluent A for 2 min, and re-equilibration with 100% eluent A for 3 min. Data were analysed with the Agilent OpenLAB CDS ChemStation (Edition C.01.04).

Simultaneous analysis of picolinic acid and quinolinic acid was performed as described previously [38] with minor modifications using an Agilent 7890A gas chromatography (GC) system coupled with Agilent 5975 C mass spectrometry detector and Agilent 7693A autosampler (Agilent Technologies, Inc., Santa Clara, CA) with 1 μL of derivatized mixture. Separation of picolinic acid and quinolinic acid was achieved with a DB-5MS column, 0.25 μm film thickness, and 0.25 mm × 30 m capillary column (Agilent Technologies, Inc., Santa Clara, CA) within 7 min, but the assay run time was set for 12 min to prevent sample carryover. Picolinic acid and quinolinic acid concentrations were analysed using Agilent GC/MSD ChemStation software (Edition 02.02.1431) and interpolated from the established 6-point calibration curves based on the abundance count ratio of the metabolites to their corresponding deuterated internal standards within each standard and sample. The intra- and inter-assay CV was within the acceptable range (4–8% for UHPLC assays, 7–10% for GCMS assays) calculated from the repeated measures of the metabolite standards incorporated during the sequence run.

### Neuroimaging

All study participants were imaged within 3 months of blood collection. Participants underwent positron emission tomography (PET) using ligand ^18^F-florbetaben at Macquarie Medical Imaging in Sydney. Participants were administered an intravenous bolus of ^18^F-florbetaben slowly over 30 s, while in a rested position. Images were acquired over a 20-min scan, in 5 min acquisitions, beginning 50 min post-injection. Neocortical amyloid-β load was calculated as the mean standard uptake value ratio (SUVR) of the frontal, superior parietal, lateral temporal, lateral occipital, and anterior and posterior cingulate regions with the cerebellum as the reference region, using image processing software CapAIBL [39, 40]. Study participants with an SUVR< 1.35 were categorised as low NAL, while those with an SUVR ≥ 1.35 were classified as high NAL.

### Statistical analyses

Descriptive statistics including means and standard deviations were calculated for NAL− and NAL+ groups (Table 1) and for NFL quartiles Q1 vs. Q2, Q3, and Q4 (Additional file 1: Table S1). Chi-square tests were employed to compare the frequency of gender and *APOE* ε4 carrier status between NAL− and NAL+ groups, and across NFL quartiles. Additionally, linear models were employed to compare continuous variables examined between study groups. Continuous response variables were tested for approximate normality and log transformed when required to satisfy test criteria. Pearson’s correlation coefficient was employed to investigate correlations. Partial correlations were used when associations investigated were adjusted for age, gender, and *APOE* ε4 carrier status. All analyses were carried out using IBM® SPSS® version 23.

**Table 1:** Cohort characteristics

	All participants	Low NAL	High NAL	*p*
Gender (*N*, males/females)	32/68	19/46	13/22	.419
Age (years)	78.18 ± 5.52	77.61 ± 5.55	79.22 ± 5.38	.165
*APOE* ε4 carriers (*N*, %)	21 (21)	5 (7.7)	16 (45.7)	< .0001
Education (years)	14.43 ± 3.26	14.84 ± 3.37	13.64 ± 2.91	.078
MMSE (score)	28.61 ± 1.14	28.50 ± 1.16	28.80 ± 1.10	.225
NAL (SUVR)	1.35 ± 0.31	1.15 ± 0.08	1.71 ± 0.26	–
Tryptophan (μM)	43.33 ± 7.72	42.87 ± 8.15	44.17 ± 6.87	.350
Kynurenine (μM)	2.23 ± 0.58	2.12 ± 0.52	2.41 ± 0.63	< .05
Kynurenine to tryptophan ratio (K/T)	52.61 ± 15.44	50.73 ± 13.94	56.08 ± 17.58	.121
Kynurenic Acid (nM)	52.06 ± 27.44	49.71 ± 26.25	56.41 ± 29.40	.238
3-Hydroxykynurenine (nM)	119.45 ± 37.47	117.74 ± 34.73	122.60 ± 42.42	.554
3-Hydroxyanthranilic acid (nM)	22.63 ± 9.14	22.15 ± 9.25	23.50 ± 8.99	.406
Anthranilic acid (nM)	35.48 ± 19.79	31.40 ± 14.99	43.04 ± 25.02	< .005
Picolinic acid (nM)	109.38 ± 49.82	112.06 ± 56.25	104.40 ± 35.07	.572
Quinolinic acid (nM)	171.83 ± 74.48	169.76 ± 75.89	175.66 ± 72.71	.689

Baseline characteristics including gender, age, *APOE ε4* status, education, Mini-Mental State Examination (MMSE) scores, and neocortical amyloid-β load (NAL) represented by the standard uptake value ratio (SUVR) of ligand ^18^F-florbetaben assessed via positron emission tomography, in the neocortical region normalised with that in the cerebellum, have been compared between low NAL (SUVR< 1.35) and high NAL (SUVR ≥ 1.35) study participants. Chi-square tests or linear models were employed as appropriate. Data have been presented in mean ± SD unless otherwise mentioned. Kynurenine to tryptophan ratios for all participants were multiplied by 1000. All *p* values for the kynurenine pathway metabolites were obtained from variables transformed to the logarithmic scale for analyses to meet assumptions of the statistical test employed

## Results

### Cohort characteristics

Cohort characteristics are presented in Table 1. The cohort was divided into low and high NAL groups or grouped according to NFL quartiles (Additional file 1: Table S1). On comparing the low NAL with the high NAL group, no significant differences were observed, except for the significantly higher frequency of *APOE* ε4 carriers within the high NAL group, which was expected given that carriage of the *APOE* ε4 allele is a known risk factor for AD and high NAL [41]. Higher age and lower MMSE scores were associated with higher NFL quartiles as expected [42]. No significant differences in education and NAL were observed between individuals categorised based on their plasma NFL concentrations. Additionally, no significant differences in gender or *APOE* ε4 carrier status were observed between individuals lying in the different NFL quartiles.

### Correlation between NFL and KP metabolites in all participants

**Fig. 2:**
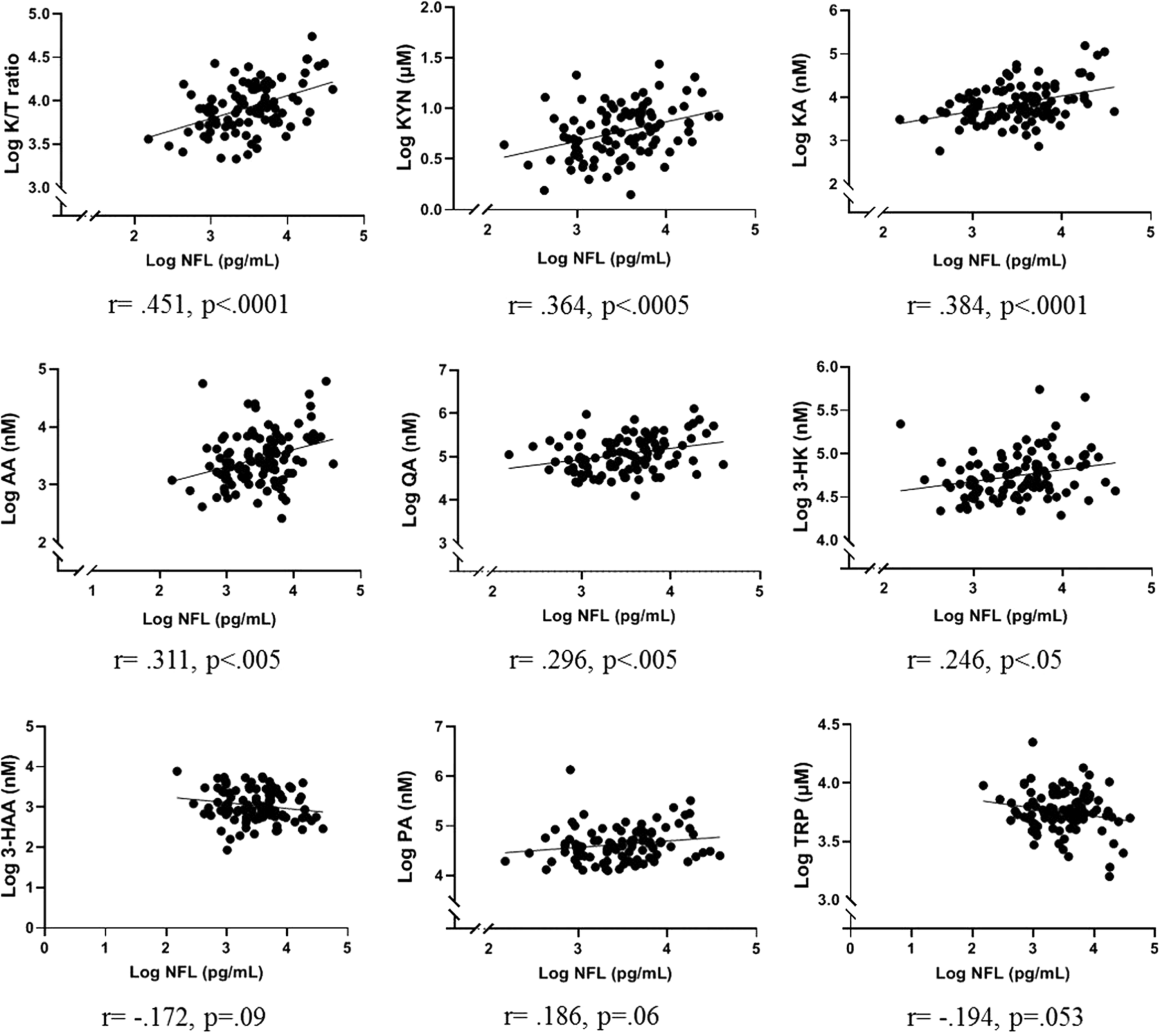
Correlations between KP metabolites and NFL in all participants. Plasma neurofilament light chain (NFL) correlated with the kynurenine to tryptophan ratio (K/T) and other kynurenine pathway (KP) metabolites, namely kynurenine (KYN), kynurenic acid (KA), 3-hydroxykynurenine (3-HK), anthranilic acid (AA), and quinolinic acid (QA) using Pearson’s correlation coefficient. Log transformed plasma NFL and KP analyte concentrations have been presented

NFL was observed to have a positive correlation with the K/T ratio (*r* = .451, *p* < .0001). Positive correlations were also observed between NFL and KP metabolites, KYN (*r* = .364, *p* < .0005), KA (*r* = .384, *p* < .0001), 3-HK (*r* = .246, *p* = .014), AA (*r* = .311, *p* = .002), and QA (*r* = .296, *p* = .003). Figure 2 presents correlations between NFL and KP metabolites. After adjusting for age, gender, and *APOE* ε4 status, all the above-mentioned associations between NFL and KP metabolites, except for 3-HK, continued to remain significant (Additional file 2: Table S2).

### Correlation between NFL and KP metabolites in participants with low NAL

**Fig. 3:**
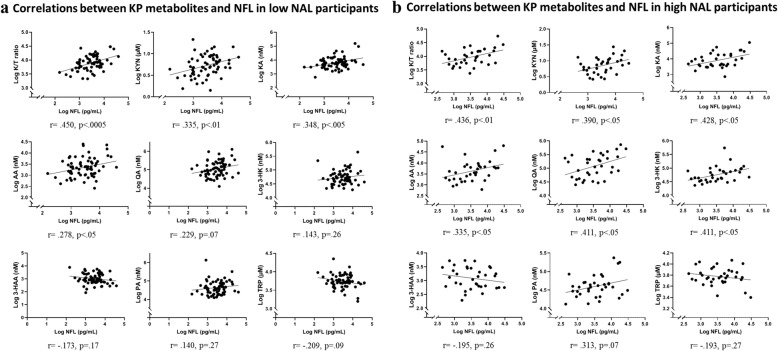
Correlations between KP metabolites and NFL in participants with low NAL (**a**) and high NAL (**b**). Plasma neurofilament light chain (NFL) correlated with the kynurenine to tryptophan ratio (K/T) and other kynurenine pathway (KP) metabolites, namely kynurenine (KYN), kynurenic acid (KA), and anthranilic acid (AA) in participants with low NAL. Plasma NFL correlated with the K/T and other kynurenine pathway KP metabolites, namely KYN, KA, AA, 3-HK, and QA. Analyses were carried out using Pearson’s correlation coefficient. Log transformed plasma NFL and KP analyte concentrations have been presented

NFL positively correlated with the K/T ratio (*r* = .450, *p* < .0005) in the 65 participants with low NAL. Positive correlations were also observed between NFL and KP metabolites, KYN (*r* = .335, *p* = .006), KA (*r* = .348, *p* = .005), and AA (*r* = .278, *p* = .025). Figure 3 presents correlations between NFL and KP metabolites. After adjusting for age, gender, and *APOE* ε4 status, the above-mentioned associations between NFL and KP metabolites continued to remain significant (Additional file 2: Table S2).

### Correlation between NFL and KP metabolites in participants with high NAL

NFL positively correlated with the K/T ratio (*r* = .436, *p* = .009) in the 35 participants with high NAL. Positive correlations were also observed between NFL and KP metabolites, KYN (*r* = .390, *p* = .020), KA (*r* = .428, *p* = .010), 3-HK (*r* = .411, *p* = .014), AA (*r* = .335, *p* = .049), and QA (*r* = .411, *p* = .014). Figure 3 presents correlations between NFL and KP metabolites. After adjusting for age, gender, and *APOE* ε4 status, KYN and KA remained significant (Additional file 2: Table S2).

### Correlation between plasma Aβ and KP metabolites in all participants

**Fig. 4:**
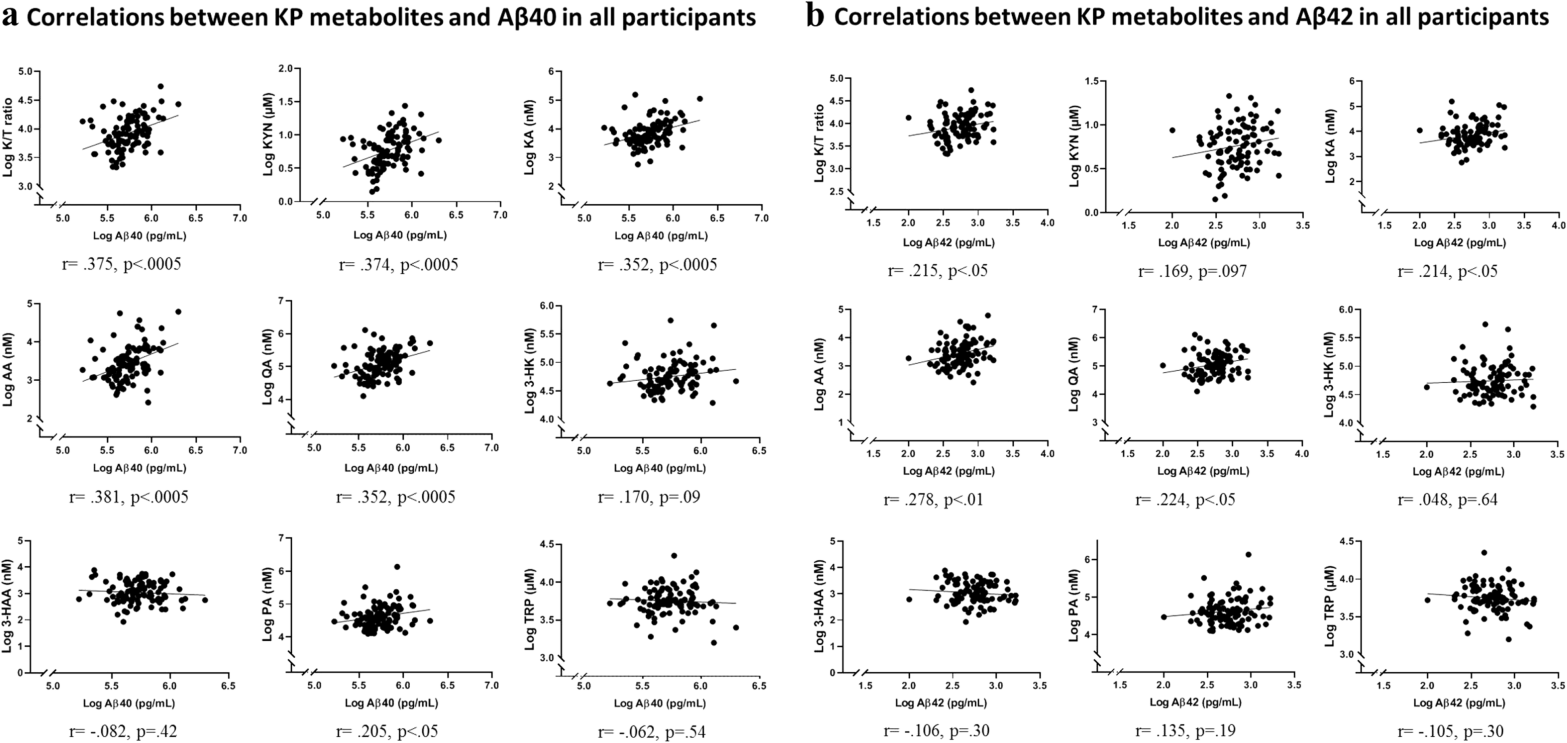
Correlations between KP metabolites and Aβ40 (**a**) and Aβ42 (**b**) species in all participants. Plasma Aβ40 correlated with the kynurenine to tryptophan ratio (K/T) and other kynurenine pathway (KP) metabolites, namely kynurenine (KYN), kynurenic acid (KA), anthranilic acid (AA), quinolinic acid (QA), and picolinic acid (PA) using Pearson’s correlation coefficient. Plasma Aβ42 correlated with the kynurenine to tryptophan (K/T) ratio and other kynurenine pathway (KP) metabolites, namely kynurenic acid (KA), anthranilic acid (AA), and quinolinic acid (QA) using Pearson’s correlation coefficient. Log transformed plasma Aβ and KP analyte concentrations have been presented

Plasma Aβ40 positively correlated with the K/T ratio (*r* = .375, *p* < .0005). A positive correlation was also observed between Aβ40 and KP metabolites, KYN (*r* = .374, *p* < .0005), KA (*r* = .352, *p* < .0005), AA (*r* = .381, *p* < .0005), PA (*r* = .205, *p* = .042), and QA (*r* = .352, *p* < .0005). Figure 4 presents correlations between Aβ40 and KP metabolites. After adjusting for age, gender, and *APOE* ε4 status, all the above-mentioned associations between Aβ40 and KP metabolites, except for PA, continued to remain significant (Additional file 3: Table S3). Plasma Aβ42 positively correlated with the K/T ratio (*r* = .215, *p* = .034), KA (*r* = .214, *p* = .035), AA (*r* = .278, *p* = .006), and QA (*r* = .224, *p* = .027). Figure 4 presents correlations between Aβ42 and KP metabolites. After adjusting for age, gender, and *APOE* ε4 status, Aβ42 only continued to correlate significantly with KA and AA while a trend remained for QA (Additional file 4: Table S4). Furthermore, the plasma Aβ40/42 ratio correlated with KYN (*r* = .236, *p* = .024) after adjusting for age, gender, and *APOE* ε4 status.

### Correlation between plasma Aβ and KP metabolites in participants with low NAL

**Fig. 5:**
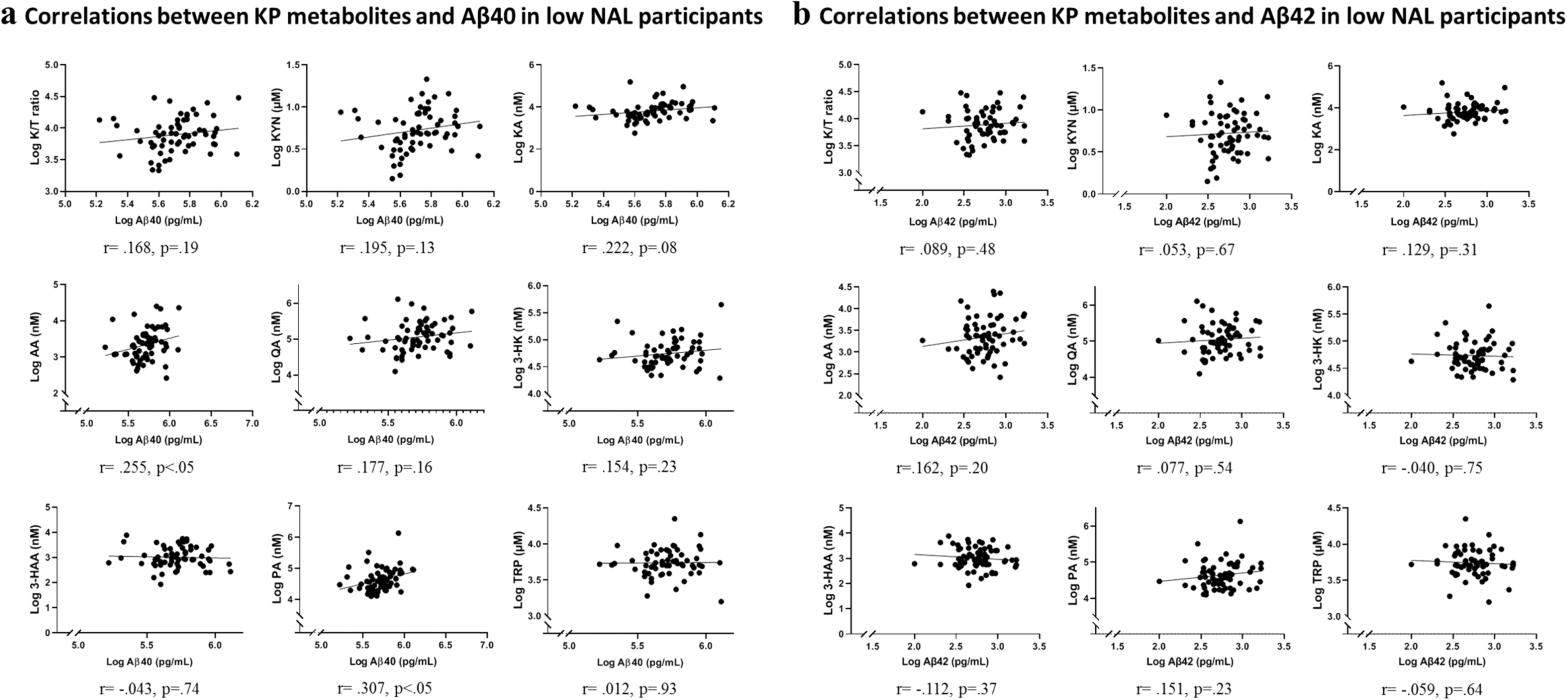
Correlations between KP metabolites and Aβ40 (**a**) and Aβ42 (**b**) in participants with low NAL. Plasma Aβ40 correlated with anthranilic acid (AA) and picolinic acid (PA) using Pearson’s correlation coefficient. Log transformed plasma Aβ and KP analyte concentrations have been presented

Plasma Aβ40 positively correlated with AA (*r* = .255, *p* = .044) and PA (*r* = .307, *p* = .014) while plasma Aβ42 was not observed to correlate with any KP metabolite in low NAL (*n* = 63 for Aβ40, *n* = 65 for Aβ42) participants. After adjusting for age, gender, and *APOE* ε4 status, only PA correlated with Aβ40 (Additional file 3: Table S3). Figure 5 presents correlations between Aβ40 and KP metabolites. The plasma Aβ40/42 ratio correlated with 3-HK (*r* = .255, *p* = .049) after adjusting for age, gender, and *APOE* ε4 status.

### Correlation between plasma Aβ and KP metabolites in participants with high NAL

**Fig. 6:**
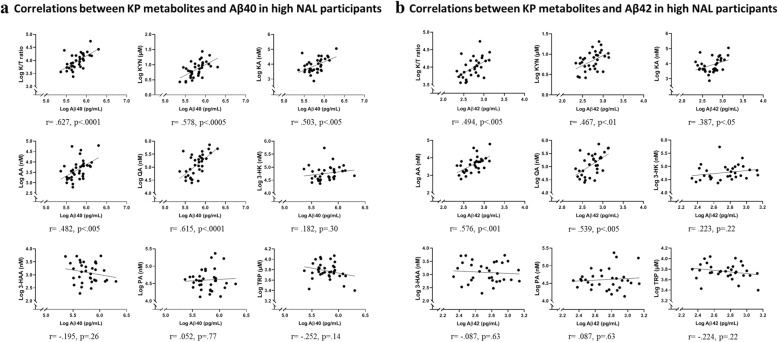
Correlations between KP metabolites and Aβ40 (**a**) and Aβ42 (**b**) species in participants with high NAL. Plasma Aβ40 correlated with the kynurenine to tryptophan ratio (K/T) and other kynurenine pathway (KP) metabolites, namely kynurenine (KYN), kynurenic acid (KA), anthranilic acid (AA), and quinolinic acid (QA) using Pearson’s correlation coefficient. Plasma Aβ42 correlated with the kynurenine to tryptophan ratio (K/T) and other kynurenine pathway (KP) metabolites, namely kynurenine (KYN), kynurenic acid (KA), anthranilic acid (AA), and quinolinic acid (QA) using Pearson’s correlation coefficient. Log transformed plasma Aβ and KP analyte concentrations have been presented

Plasma Aβ40 positively correlated with the K/T ratio (*r* = .627, *p* < .0001) in participants with high NAL. A positive correlation was also observed between Aβ40 and KP metabolites, KYN (*r* = .578, *p* < .0005), KA (*r* = .503, *p* = .002), AA (*r* = .482, *p* = .003), and QA (*r* = .615, *p* < .0001) (*n* = 35 for Aβ40). Figure 6 presents correlations between Aβ40 and KP metabolites. After adjusting for age, gender, and *APOE* ε4 status, all the above-mentioned associations between Aβ40 and KP metabolites continued to remain significant (Additional file 3: Table S3). Further, plasma Aβ42 positively correlated with the K/T ratio (*r* = .494, *p* = .004), KYN (*r* = .467, *p* = .007), KA (*r* = .387, *p* = .029), AA (*r* = .576, *p* = .0006), and QA (*r* = .539, *p* = .001) (*n* = 32 for Aβ42). Figure 6 presents correlations between Aβ42 and KP metabolites. After adjusting for age, gender, and *APOE* ε4 status, Aβ42 continued to correlate significantly with the aforementioned metabolites while a trend was observed with the K/T ratio (Additional file 4: Table S4). Furthermore, the plasma Aβ40/42 ratio correlated with the K/T ratio (*r* = .462, *p* = .012) and KYN (*r* = .390, *p* = .037) after adjusting for age, gender, and *APOE* ε4 status.

## Discussion

The current study investigated the association of the KP metabolites with two emerging blood biomarkers for AD, namely NFL and Aβ. NFL has widely been reported to increase with the severity of neurodegeneration and cognitive impairment, including that in AD [8, 42–44], while plasma Aβ has shown strong potential in reflecting brain Aβ burden showing approximately 90% accuracy [29, 30].

Within the current study, significant positive associations between KP metabolites and NFL were observed. Interestingly, the positive association between the K/T ratio and NFL also suggests a positive association between IDO (the first-rate limiting enzyme of the KP) activity and NFL. Given that increased IDO activity has been observed in inflammatory conditions [25], the association observed between IDO activity (K/T ratio) and NFL within cognitively normal elderly individuals in the current study may further reflect an association between the extent of inflammation and the extent of underlying neurodegeneration. Interestingly, NFL correlated with the KP metabolites irrespective of the AD-related pathology (NAL) status in cognitively normal elderly individuals that were at no apparent risk of AD and in those with preclinical AD, characterised by high NAL. These observations perhaps reflect previous observations of elevated NFL not being specifically associated with NAL pathology but rather being common to the extent of neurodegeneration [7, 8, 45, 46].

For example, Mattsson and colleagues reported no significant differences in plasma NFL concentrations between Aβ-negative and Aβ-positive (based on CSF Aβ42) control individuals [8]. Further, no significant differences were observed in CSF NFL concentrations between Aβ-negative and Aβ-positive individuals within the control, MCI, or AD groups [45]. Additionally, a study within the autosomal dominant form of AD reported that while the rate of change of serum NFL was significantly higher in mutation carriers compared to noncarriers, the positive association observed between the rate of change of serum NFL and rate of change of brain Aβ deposition did not reach significance [46]. Findings from our study are further supported by elevated blood (and CSF) NFL in primary tauopathies with no Aβ, such as progressive supranuclear palsy, corticobasal degeneration, and multiple system atrophy [7]. Together, these findings indicate that NFL concentrations are not driven by NAL and that blood NFL is primarily a marker of neurodegeneration, which likely resulted in the observation of NAL status-independent associations between KP metabolites and NFL.

NFL together with neurofilament medium chain and neurofilament heavy chain constitutes the three neurofilament isoforms. While the neurofilaments are synthesised in the cell body, it has been posited that they are phosphorylated after transportation to the axon [47]. In neurodegenerative conditions, the phosphorylated forms of neurofilaments are observed in the cell body and the proximal region of the axon, when a disequilibrium in the phosphorylation-dephosphorylation balance of neurofilaments occurs, as opposed to the distal region of the axon, in normal conditions [48]. Interestingly, evidence shows that the KP metabolite, QA, can potentially disrupt the homeostasis of the neural cell cytoskeleton via aberrant phosphorylation, contributing to aberrant cell functioning and neurodegeneration [49].

QA has been reported to activate the *N*-methyl-d-aspartate (NMDA) receptor resulting in Ca^2+^ ion influx into the cells, activating phosphorylation enzymes and therefore resulting in phosphorylation of the cytoskeleton components [49]. An acute QA infusion in the striatum of 30-day-old rats stimulated NFL hyperphosphorylation within 30 min, associated with cAMP-dependent protein kinase A and protein kinase Ca2+/calmodulin-dependent protein kinase II activity, reflecting the vulnerability of both neuronal and astrocyte cytoskeleton to QA toxicity [49]. While these results show that QA is toxic to the cytoskeleton and may be associated with neurodegeneration, findings from the current study, most interestingly, suggest that other KP metabolites upstream to QA are also associated with the disruption of the neuronal cytoskeleton protein NFL. Whether the other KP metabolites are associated with plasma NFL via mechanisms similar to QA are yet to be investigated.

**Fig. 7:**
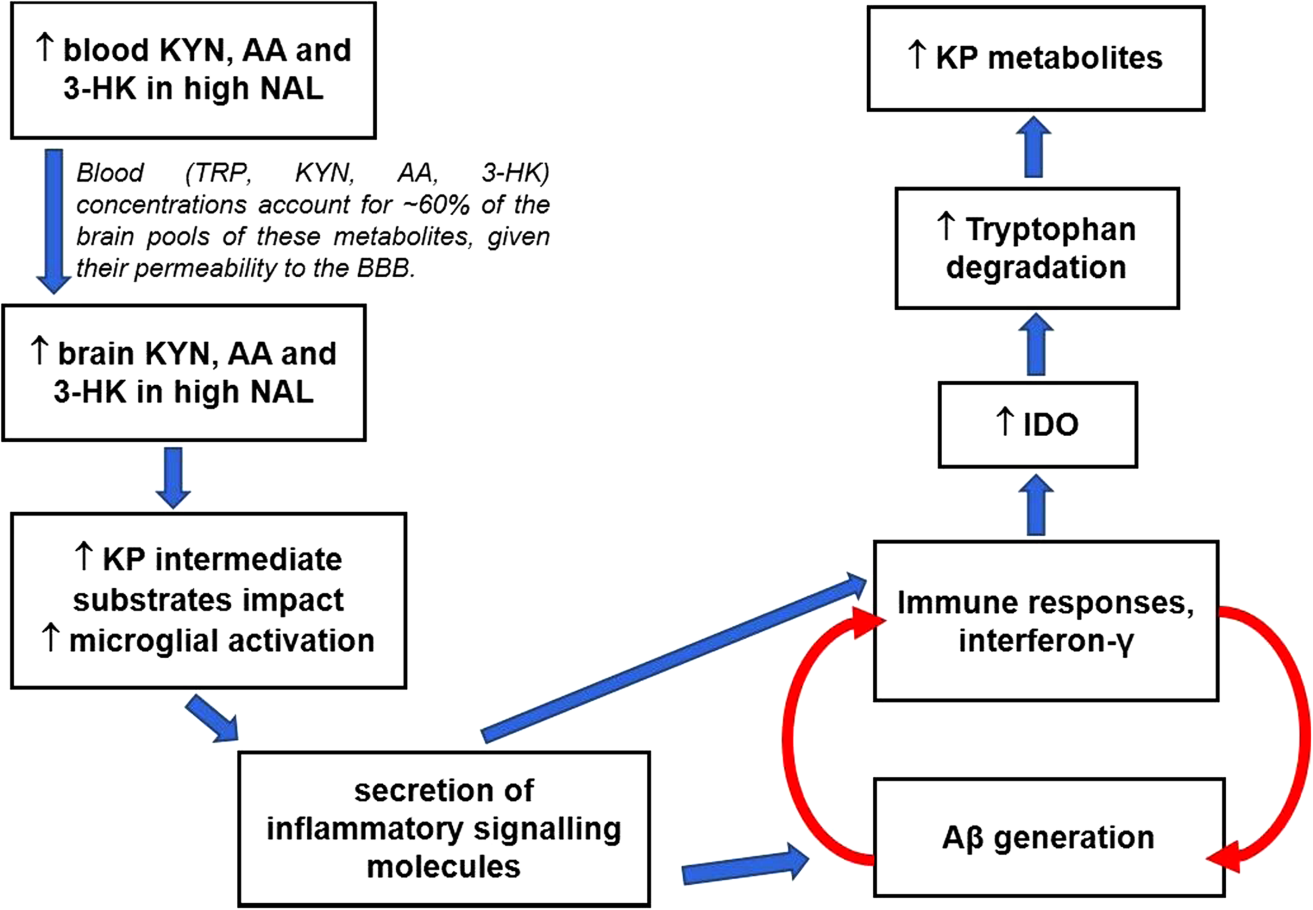
Possible mechanisms involved in the association between blood KP metabolites and Aβ in high NAL participants. Elevated blood kynurenine (KYN), anthranilic (AA), and 3-hydroxykynurenine (3-HK) concentrations in individuals with high neocortical amyloid-β load (NAL) [14] potentially have increased KYN, AA, and 3-HK concentrations in the brain, given that blood KYN, AA, and 3-HK concentrations account for ~ 60% of the brain pools of these metabolites, given their permeability to the blood-brain barrier [50]. This increase in the kynurenine pathway (KP) intermediate substrates could result in increased microglial activation which in turn can result in the secretion of inflammatory signalling molecules, which further trigger amyloid-β generation and immune responses, resulting in a vicious cycle. Further, immune responses such as interferon-γ activate indoleamine 2,3-dioxygenase result in increased tryptophan degradation and increased KP metabolites

In contrast to the similar associations observed between KP metabolites and NFL in both low and high NAL groups, the association between KP metabolites and plasma Aβ varied between the two groups, wherein associations between KP metabolites and plasma Aβ were nearly absent (particularly with K/T that reflects IDO activity) in individuals at no apparent risk of AD (or low NAL) but were pronounced between KP metabolites and plasma Aβ in preclinical AD, characterised by high NAL. These observations may be attributed to the vicious nature of the relationship between blood and brain Aβ and KP metabolites in individuals with high NAL, as described in Fig. 7.

Further, since we observed an interaction effect of NAL and gender on the KP metabolites previously [14], we also investigated whether an interaction effect of plasma Aβ and gender was present on the KP metabolites in the current study. Interestingly, no interaction effect was observed between plasma Aβ (40 or 42) and gender on KP metabolites.

It is acknowledged that the present study has its limitations with regard to its relatively modest sample size. Interestingly, unlike the associations between KP metabolites and NFL, the association between KP metabolites and the two plasma Aβ species 40 and 42 was distinct between low NAL and high NAL groups. The association between KP metabolites and plasma Aβ could be dependent on NAL such that the strong correlations between plasma Aβ and KP metabolites in the high NAL group may suggest an association between Aβ metabolism and inflammation within amyloid-positive individuals. However, the low Aβ concentrations in the plasma in the low NAL group may also contribute to these observations, although the assay used to measure plasma Aβ in the current study is an ultra-sensitive assay. Further, while more sensitive assays may aid increase Aβ measurement accuracy, the current observations also require validation in an independent cohort.

## Conclusion

The major findings of the study are that positive correlations exist between KP metabolites and plasma NFL and Aβ concentrations in elderly individuals, wherein the associations between KP metabolites and plasma NFL are NAL status independent, while the associations between KP metabolites and plasma Aβ species (40 and 42) were NAL status dependent. The peripheral concentrations of NFL, Aβ, and KP metabolites potentially reflect ongoing pathological processes in the brain. Plasma NFL levels reflect axonal cytoskeleton damage [51–53], and plasma Aβ has been shown to reflect brain Aβ deposition [29, 30], both of which contribute to neurodegeneration, while increased KP activity is indicative of neuroinflammation [16]. Since observations from the current study suggest that the association of neurodegeneration and amyloid pathology with neuroinflammation manifests in the periphery, preventing cytoskeleton cytotoxicity by elevated KP metabolites in the periphery may have therapeutic potential. Therefore, further studies on the enzymes that catalyse the production of various KP metabolites and potential KP enzyme inhibitors as potential therapeutic agents need to be investigated [54].

## Additional files

Additional file 1: **Table S1.** Cohort characteristics based on neurofilament light chain quartiles. (DOCX 15 kb)

Additional file 2: **Table S2.** Correlation between KP metabolites and NFL in all participants and after stratifying by NAL status (low/high NAL), adjusting for age, gender and APOE ε4 status. (DOCX 16 kb)

Additional file 3: **Table S3.** Correlation between plasma KP metabolites and Aβ40 in all participants and after stratifying by NAL status (low/high NAL), adjusting for age, gender and APOE ε4 status. (DOCX 16 kb)

Additional file 4: **Table S4.** Correlation between plasma KP metabolites and Aβ42 in all participants and after stratifying by NAL status (low/high NAL), adjusting for age, gender and APOE ε4 status. (DOCX 18 kb)

## Data Availability

The datasets used and/or analysed during the current study are available from the corresponding author on reasonable request.

## Abbreviations

3-HAA: 3-Hydroxyanthranilic acid
3-HK: 3-Hydroxykynurenine
AA: Anthranilic acid
ACMA: Aminocarboxymuconic semialdehyde
AD: Alzheimer’s disease
APOE: Apolipoprotein gene
Aβ: Amyloid-beta
CSF: Cerebrospinal fluid
GC: Gas chromatography
IDO: Indoleamine 2,3-dioxygenase
K/T: Kynurenine to tryptophan ratio
KA: Kynurenic acid
KARVIAH: Kerr Anglican Retirement Village Initiative in Ageing Health
KP: Kynurenine pathway
KYN: Kynurenine
MCI: Mild cognitive impairment
MMSE: Mini-Mental State Examination
MoCA: Montreal Cognitive Assessment
NAL: Neocortical amyloid-beta load
NFL: Neurofilament light chain
PA: Picolinic acid
PET: Positron emission tomography
QA: Quinolinic acid
QC: Quality control
Simoa: Single molecule array
SUVR: Standard uptake value ratio
TRP: Tryptophan
UHPLC: Ultra-high-performance liquid chromatography
