# Supplementary material for: Plasma neurofilament light chain and amyloid-β are associated with the kynurenine pathway metabolites in preclinical Alzheimer’s disease
Source: J Neuroinflammation. 2019 Oct 10;16:186. doi: 10.1186/s12974-019-1567-4 (PMC6788092; doi:10.1186/s12974-019-1567-4)
Supplement: Supplementary file 1 — Table S1. Cohort characteristics based on neurofilament light chain quartiles. (DOCX 15 kb) [file 12974_2019_1567_MOESM1_ESM.docx]

**Additional file 1: Table S1. Cohort characteristics based on neurofilament light chain quartiles.**

|  | **All participants** | **NFL Q1** | **NFL Q2** | **NFL Q3** | **NFL Q4** | **p** |
| --- | --- | --- | --- | --- | --- | --- |
| **N** | 100 | 25 | 25 | 25 | 25 | - |
| **Gender (M/F)** | 32/68 | 7/18 | 9/16 | 6/19 | 10/15 | .607 |
| **Age (years, mean ±SD)** | 78.18±5.52 | 74±4.74 | 77.12±5.59* | 80.16±4.14* | 81.44±4.51* | **8.67E-7** |
| **n*APOE*** **ε4 carriers (%)** | 21 (21) | 8 (32) | 4 (16) | 5 (20) | 4 (16) | .459 |
| **Education (years, mean ±SD)** | 14.43±3.26 | 14.96±2.95 | 13.72±3.78 | 14.28±3.57 | 14.74±2.67 | .553 |
| **MMSE (mean ±SD)** | 28.61±1.14 | 29.16±1.03 | 28.76±1.09 | 28.12±1.09* | 28.40±1.15* | **.007** |
| **NAL SUVR (mean ±SD)** | 1.35±0.31 | 1.26±0.26 | 1.37±0.33 | 1.38±0.35 | 1.40±0.31 | .369 |

“*” represents a significant difference between Q1 vs Q2, Q3, Q4.
