# Supplementary material for: Plasma neurofilament light chain and amyloid-β are associated with the kynurenine pathway metabolites in preclinical Alzheimer’s disease
Source: J Neuroinflammation. 2019 Oct 10;16:186. doi: 10.1186/s12974-019-1567-4 (PMC6788092; doi:10.1186/s12974-019-1567-4)
Supplement: Supplementary file 2 — Table S2. Correlation between KP metabolites and NFL in all participants and after stratifying by NAL status (low/high NAL), adjusting for age, gender and APOE ε4 status. (DOCX 16 kb) [file 12974_2019_1567_MOESM2_ESM.docx]

**Additional file 2: Table S2. Correlation between KP metabolites and NFL in all participants and after stratifying by NAL status (low/high NAL), adjusting for age, gender and APOE ε4 status**

| Plasma NFL (pg/mL) | K/T ratio | KYN  µM | KA  nM | AA  nM | QA  nM | 3-HK  nM | 3-HAA  nM | PA  nM |
| --- | --- | --- | --- | --- | --- | --- | --- | --- |
| All participants | r=.380  p<.0005 | r= .345  p<.001 | r= .383  p<.0005 | r= .271  p=.007 | r=.201  p=.049 | r=.188  p=.066 | r= -.137  p=.180 | r=.139  p=.175 |
| Participants with low NAL | r=.344  p=.006 | r=.283  p=.026 | r= .305  p=.016 | r= .259  p=.042 | r=.080  p=.536 | r=.063  p=.624 | r= -.195  p=.128 | r=.093  p=.475 |
| Participants with high NAL | r=.355  p=.046 | r= .392  p=.027 | r= .468  p=.007 | r=.158  p=.388 | r=.316  p=.078 | r=.279  p=.122 | r= -.010  p=.955 | r=.178  p=.329 |
