# Supplementary material for: Plasma neurofilament light chain and amyloid-β are associated with the kynurenine pathway metabolites in preclinical Alzheimer’s disease
Source: J Neuroinflammation. 2019 Oct 10;16:186. doi: 10.1186/s12974-019-1567-4 (PMC6788092; doi:10.1186/s12974-019-1567-4)
Supplement: Supplementary file 3 — Table S3. Correlation between plasma KP metabolites and Aβ40 in all participants and after stratifying by NAL status (low/high NAL), adjusting for age, gender and APOE ε4 status. (DOCX 16 kb) [file 12974_2019_1567_MOESM3_ESM.docx]

**Additional file 3: Table S3. Correlation between plasma KP metabolites and Aβ40 in all participants and after stratifying by NAL status (low/high NAL), adjusting for age, gender and APOE ε4 status**

| Plasma Aβ40 (pg/mL) | K/T ratio | KYN  µM | KA  nM | AA  nM | QA  nM | 3-HK  nM | 3-HAA  nM | PA  nM |
| --- | --- | --- | --- | --- | --- | --- | --- | --- |
| All participants | r=.336  p=.001 | r= .350  p=.001 | r= .332  p=.001 | r= .352  p<.0005 | r=.315  p=.002 | r=.142  p=.170 | r= -.081  p=.436 | r=.182  p=.077 |
| Participants with low NAL | r=.091  p=.492 | r=.139  p=.290 | r=.135  p=.303 | r=.193  p=.140 | r=.100  p=.447 | r= .097  p=.461 | r= -.099  p=.452 | r= .279  p=.031 |
| Participants with high NAL | r=.583  p<.0005 | r= .559  p=.001 | r= .508  p=.003 | r= .410  p=.020 | r=.567  p=.001 | r=.027  p=.884 | r= -.098  p=.595 | r= -.073  p=.690 |
