# Supplementary material for: Plasma neurofilament light chain and amyloid-β are associated with the kynurenine pathway metabolites in preclinical Alzheimer’s disease
Source: J Neuroinflammation. 2019 Oct 10;16:186. doi: 10.1186/s12974-019-1567-4 (PMC6788092; doi:10.1186/s12974-019-1567-4)
Supplement: Supplementary file 4 — Table S4. Correlation between plasma KP metabolites and Aβ42 in all participants and after stratifying by NAL status (low/high NAL), adjusting for age, gender and APOE ε4 status. (DOCX 18 kb) [file 12974_2019_1567_MOESM4_ESM.docx]

**Additional file 4: Table S4. Correlation between plasma KP metabolites and Aβ42 in all participants and after stratifying by NAL status (low/high NAL), adjusting for age, gender and APOE ε4 status.**

| Plasma Aβ42 (pg/mL) | K/T ratio | KYN  µM | KA  nM | AA  nM | QA  nM | 3-HK  nM | 3-HAA  nM | PA  nM |
| --- | --- | --- | --- | --- | --- | --- | --- | --- |
| All participants | r=.188  p=.069 | r=.153  p=.141 | r=.219  p=.034 | r=.272  p=.008 | r=.200  p=.054 | r=.018  p=.866 | r= -.099  p=.344 | r=.119  p=.255 |
| Participants with low NAL | r=.041  p=.754 | r=.026  p=.843 | r=.084  p=.515 | r=.131  p=.311 | r=.025  p=.848 | r= -.074  p=.568 | r= -.139  p=.280 | r=.141  p=.273 |
| Participants with high NAL | r=.362  p=.054 | r=.381  p=.041 | r=.380  p=.042 | r=.469  p=.010 | r=.439  p=.017 | r=.007  p=.970 | r=.011  p=.953 | r= -.035  p=.857 |
